# Prevalence of Acute Kidney Injury and Prognostic Significance in Patients with Acute Myocarditis

**DOI:** 10.1371/journal.pone.0048055

**Published:** 2012-10-29

**Authors:** Ya-Wen Yang, Che-Hsiung Wu, Wen-Je Ko, Vin-Cent Wu, Jin-Shing Chen, Nai-Kuan Chou, Hong-Shiee Lai

**Affiliations:** 1 Division of General Surgery, Department of Surgery, National Taiwan University Hospital, Taipei, Taiwan; 2 Division of Nephrology, Department of Medicine, Tzu Chi General Hospital Taipei Branch, Xindian District, New Taipei City, Taiwan; 3 Division of Nephrology, Department of Medicine, National Taiwan University Hospital, Taipei, Taiwan; University of Leicester, United Kingdom

## Abstract

**Objective:**

Myocarditis is an inflammation of the myocardium. The condition is commonly associated with rapid disease progression and often results in profound shock. Impaired renal function is the result of impairment in end-organ perfusion and is highly prevalent among critically ill patients. The aim of this study was to evaluate the incidence of acute kidney injury (AKI) and identify the relationship between AKI and the prognosis of patients with acute myocarditis.

**Design, Measurements and Main Results:**

This retrospective study reviewed the medical records of 101 patients suffering from acute myocarditis between 1996 and 2011. Sixty of these patients (59%) developed AKI within 48 hours of being hospitalized. AKI defined as AKIN stage 3 (p = 0.007) and SOFA score (p = 0.03) were identified as predictors of in-hospital mortality in multivariate analysis. The conditional effect plot of the estimated risk against SOFA score upon admission categorized according to the AKIN stages showed that the risk of in-hospital mortality was highest among patients in AKIN stage 3 with a high SOFA score.

**Conclusions:**

Among patients with acute myocarditis, AKI defined as AKIN stage 3 and elevated SOFA score were associated with unfavorable outcomes. AKIN classification is a simple, reproducible, and easily applied evaluation tool capable of providing objective information related to the clinical prognosis of patients with acute myocarditis.

## Introduction

Myocarditis refers to an inflammation of the myocardium which can lead to diverse clinical outcomes ranging from asymptomatic courses to cardiogenic shock. Impaired renal function is the result of impaired end-organ perfusion due to a failing heart. The condition is highly prevalent among critically ill patients. In previous studies, 33% of patients with acute decompensated heart failure were identified with AKI upon admission, but this increased to 73% during their hospital stay [1]. In the past few years, AKI has been confirmed as an independent predictor of prognosis in critically ill patients [2]–[8]. Several definitions that classify the severity of AKI currently exist in the literature. Research endorsed by the Acute Dialysis Quality Initiative led to the publication of a consensus definition for AKI–the RIFLE classification, which categorized acute renal dysfunction into three grades of increasing severity and two classes of outcome [9]. Revised versions of this classification by the Acute Kidney Injury Network (AKIN) have proven to be a useful tool in the evaluation of prognostic impact among patients who had undergone cardiac surgery [10], [11]. Until now, no clinically useful parameters have been provided to determine the prognosis of adult patients with acute myocarditis [12]. The aim of this study was to evaluate the incidence of AKI using the AKIN classification as well as identify the relationship between AKI and the relevance of prognosis provided for patients with acute myocarditis.

## Materials and Methods

### Study Population

This study used data obtained from the National Taiwan University Hospital Study Group on Acute Renal Failure database (NSARF). The database was constructed prospectively to safeguard the outcome of patients 18 years or older for the period covering July 1996 to March 2011 in one medical center (National Taiwan University Hospital in Taipei, Taiwan) and three branch hospitals in different cities [13]–[17]. Patients with chronic renal disease and those in need of regular hemodialysis were excluded. The primary study outcome was in-hospital mortality. Data related to individual identification were removed and the patients remained anonymous during the entire study. The study was approved by the Institutional Review Board of the National Taiwan University Hospital the study (No.31MD03). Informed consent was waived because there was no breach of privacy and the study did not interfere with clinical decisions related to patient care.

Demographic data such as age and gender were collected from medical records. Biochemical variable included serum creatinine, blood urea nitrogen (BUN), white blood cell (WBC) count, creatine kinase (CK), CK-MB and troponin I. Clinical parameters included the Acute Physiology and Chronic Health Evaluation II (APACHE II) score [18], Sequential Organ Failure Assessment (SOFA) score [19], inotropic equivalents (IE), admission duration, requirement of extracorporeal membrane oxygenation (ECMO), left ventricular ejection fraction (LVEF) by ultrasound examination and AKI as defined according to AKIN classification.

### Data Collection

In this study, myocarditis was diagnosed clinically according to the definition from previous reports [20]. Because the type and dosage of catecholamines preferred by physicians can vary, this study compared the dosage of catecholamines according to inotropic equivalents (IE, µg/kg/min  =  dopamine + dobutamine + 100 X epinephrine + 100 X norepinephrine + 100 X isoprotenolol + 15 X milrinone) [21] in order to compare the severity of heart failure. Patients who experienced profound, rapidly progressive ventricular dysfunction with persistently low blood pressure (systolic blood pressure <80 mm Hg for adults), as measured by arterial lines and oliguria (<0.5 ml/kg/hour) for at least 4 hours under IE >40 µg/kg/min support, were considered for ECMO intervention.

The occurrence of AKI was categorized into three stages of severity (Stage 1, 2 and 3) according to AKIN classification (Table 1). Patients requiring renal replacement therapy (RRT) within the 48 hours of being hospitalized were classified as AKIN stage 3, irrespective of their serum creatinine levels and urine output.

**Table 1:** Acute Kidney Injury Network classification of acute kidney injury.

Stage	Serum creatinine criteria	Urine output criteria
1	Increase in serum creatinine of more than or equal to 0.3 mg/dl (≥26.4 µmol/l) or increaseto more than or equal to 150% to 200% (1.5- to 2-fold) from baseline	Less than 0.5 ml/kg per hour for more than 6 hours
2	Increase in serum creatinine to more than 200% to 300% (>2- to 3-fold) from baseline	Less than 0.5 ml/kg per hour for more than 12 hours
3	Increase in serum creatinine to more than 300% (>3-fold) from baseline (or serum creatinineof more than or equal to 4.0 mg/dl [≥354 µmol/l] with an acute increase of at least0.5 mg/dl [44 µmol/l])	Less than 0.3 ml/kg per hour for 24 hours or anuria for 12 hours

Acute kidney injury defined as an abrupt reduction in kidney function within 48 hours.

Individuals who receive renal replacement therapy are considered to have met the criteria for stage 3.

### Statistical Analysis

Descriptive statistics for categorical variables were expressed as frequency and percentage while continuous variables were reported as mean ± standard deviation or median (interquartile range) as appropriate. Comparisons of AKI patients according to their AKIN classification were based on chi-square test or Fisher’s exact test for categorical data; while one-way ANOVA was used for comparing age (for normal assumption assumed) and Kruskal Wallis test was used for the other continuous variables.

Logistic regression analysis with stepwise variable selection was applied for the evaluation of in-hospital mortality. Risk factors with p<0.05 (according to univariate analysis) were included in the multivariate logistic stepwise regression. Goodness of fit (GOF) was tested using the Hosmer-Lemeshow test in order to determine the accuracy of calibration. Discriminative power was assessed using the area under a receiver operating characteristic (AUROC) curve. The adjusted generalized R2 for logistic regression model is usually low. Large p-values of the Hosmer-Lemeshow GOF test indicated better fits. Conditional effect plots for the prediction of outcome were drawn based on the fitted final logistic regression model in order to plot the estimated probability of having an unfavorable outcome against a chosen continuous covariate, with the values of the other discrete and continuous covariates held constant.

## Results

### Patient Characteristics and Incidence of AKI

All of the 101 adult patients in this study were free from chronic renal insufficiency. The mean age was 39±14.8 years and 54 patients (54%) were male. Endomyocardial biopsy (EMB) was performed on 65 patients (64%) and myocarditis was proven in 33 patients (51% of patients underwent a biopsy). The median length of stay in hospital was 15 days (range 9.5 to 32.5 days). Disease severity was determined using APACHE II (median score was 12, range 7.5 to 16.5) and SOFA (median score was 8, range 4.5 to 11.0) scores. According to AKIN classification, 60 patients (59%) developed AKI within 48 hours of being hospitalized. The incidence of AKI stage 1, stage 2 and stage 3 was 17% (n = 17), 5% (n = 5) and 38% (n = 38), respectively. Among the patients with AKI, higher severity score and cardiac enzyme levels were associated with a higher degree of renal dysfunction (Table 2).

**Table 2:** Variables of different study groups of AKIN classification.

Characteristic	No-AKI (n = 41)	AKIN stages 1 and 2 (n = 22)	AKIN stage 3 (n = 38)	p-value
**Demographic data**				
Age (year)	36.2±14.4	43.9±17.2	39.1±13.2	0.2
Gender (male)	25(61)	11(50)	14(36)	0.1
**Biochemical data**				
Serum creatinine (mg/dL)	1.1 (0.9–1.2)	1.6 (1.2–1.9)	1.4 (1.0–2.2)	0.0008
BUN (mg/dL)	15.8 (12.2–23.5)	23 (14.3–34.3)	27.4 (20.3–42.5)	0.004
WBC count (10^3^/uL)	10.6 (8–14.9)	12.6 (8.2–15.3)	10.1 (7.5–14.1)	0.9
CK (U/L)	550 (339–1214)	611 (331–1325)	1376 (777–2034)	0.003
CK-MB (U/L)	35.5 (25.9–78.8)	49.7 (25–93.2)	125.9 (56.0–187.3)	<0.0001
Troponin I (ng/ml)	10.3 (0.7–21.2)	19.7 (0.7–37)	38.6 (13.5–80.0)	0.001
**Clinical parameters**				
Admission duration (days)	12 (8–19)	19 (13–43)	24.5 (7.0–39.0)	0.01
APACHE score	7.2 (0–28)	13.4 (2–30)	16 (5–27)	<0.0001
SOFA score	4.0 (2–8)	8.5 (6–12)	10.0 (8–12)	<0.0001
Log IE	0.2 (−1–1.3)	1.5 (1–1.8)	1.5 (1.0–1.7)	<0.0001
ECMO support	18 (44)	16 (73)	38 (100)	<0.0001
LVEF (%)	39 (27–53.8)	30.5 (26.1–39)	25.0 (18.0–38.0)	0.007
In-hospital mortality	2 (5)	5 (23)	22 (58)	<0.0001

Descriptive statistics for categorical variables were expressed as frequency and percentage while continuous variables were reported as mean ± standard deviation or median (interquartile range) as appropriate.

### In-hospital Mortality and Impact of AKI

Overall in-hospital mortality was 29% (n = 29). Univariate analysis revealed that BUN (p* = *0.02), CK-MB (p* = *0.05), troponin I (p* = *0.04), APACHE II score (p* = *0.002), SOFA score (p*<*0.0001), logIE (p* = *0.03), LVEF (p = 0.01) and AKIN classification were all associated with in-hospital mortality. Furthermore, AKI defined as AKIN stage 3 and elevated SOFA score were identified as independent risk factors of in-hospital mortality using multivariate logistic stepwise regression. Odds ratio was 12.3 (p = 0.007) for AKIN stage 3 versus no-AKI and rose by an increment of 1.3 (p = 0.03) for every 1-point increase in SOFA score (Table 3).

**Table 3:** Independent predictive factors in logistic regression analysis for in-hospital mortality.

	Univariate analysis		Multivariate analysis	
Variable	OR (95% CI)	p*-*value	OR (95% CI)	p-value
BUN (mg/dL)	1.0 (1.0–1.1)	0.02	1.0 (1.0–1.1)	0.6
CK-MB (U/L)	1.0 (1.0–1.007)	0.05	1.0 (1.0–1.01)	0.4
troponin I(ng/ml)	1.0 (1.001–1.03)	0.04	1.0 (1.0–1.01)	0.5
APACHE score	1.1 (1.0–1.2)	0.002	0.9 (0.8–1.028)	0.1
SOFA score	1.0 (1.1–1.5)	<0.0001	1.3 (1.0–1.7)	0.03
Log IE	1.6 (1.0–2.4)	0.03	1.3 (0.7–2.5)	0.4
LVEF (%)	1.0 (0.9–1.0)	0.01	1.0 (0.9–1.0)	0.5
Non-AKI	1		1	
AKIN stages1 and 2	5.7 (1.0–32.5)	0.05	3.5 (0.5–25.2)	0.2
AKIN stage 3	26.8 (5.6–127.6)	<0.001	12.3 (2.0–76.1)	0.007

CI, confidence interval.

**Figure 1:**
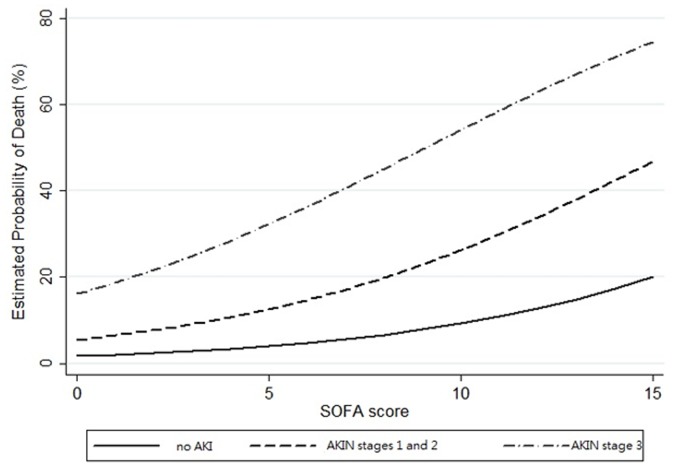
Conditional effect plot of the estimated risk for in-hospital mortality against SOFA score upon admission, stratified by AKIN stage with the values of all other factors fixed. The risk of in-hospital mortality was the highest among patients in AKIN stage 3 with a high SOFA score.

The final multiple logistic regression model exhibited very high discriminatory power (estimated area under the curve of receiver operating characteristics [eAUC-ROC] = 0.8±0.05, 95% confidence interval: 0.7–0.9). The model fit the observed binary data very well (adjusted generalized R2 = 0.4 and Hosmer-Lemeshow GOF test p = 0.4). Figure 1 presents a conditional effect plot of the estimated risk of in-hospital mortality against SOFA score upon admission, stratified according to AKIN stage with the values of all other factors fixed. The risk of in-hospital mortality was highest among patients in AKIN stage 3 with a high SOFA score.

## Discussion

In our cohort, nearly three-fifths of patients who suffered from acute myocarditis had AKI. Multivariate analysis identified SOFA score and AKI defined as AKIN stage 3 as predictors of in-hospital mortality. Some evidence suggested that a ≥25% or a ≥0.3 mg/dL increase in serum creatinine was associated with in-hospital mortality or re-hospitalization in patients suffering from heart failure [22]. In our patients, almost no difference was observed in the prognosis of patients defined as no-AKI and those in AKIN stages 1 and 2; however, AKIN stage 3 was identified as a risk factor of in-hospital mortality (p = 0.007). Diagnosis based on RIFLE classification is determined according to changes over a one-week period; whereas AKIN classification only requires changes within a designated 48-hour period. Therefore, this study employed AKIN classification to characterize the rapid progression of acute myocarditis. Because RRT may have a confounding effect on the predictive power of the AKIN classification [8], [23], we tried to re-stage patients without RRT as a criterion. Some patients originally defined as AKIN stage 3 were re-classified as other stages (5 patients were re-classified as AKIN stage 2 and 2 patients were re-classified as no-AKI). After re-staging, the AKIN stage 3 (odds ratio was 14.6 versus no-AKI, p = 0.002) was identified as a predictor of in-hospital mortality in multivariate analysis.

SOFA score was designed to describe morbidity according to 6 different scores, one each for the respiratory, cardiovascular (with adjustment for inotropic agents), hepatic, coagulation, renal, and neurological systems [19]. Various investigations have identified a clear relationship between organ dysfunction and mortality [24], [25]. In our patients, an elevated SOFA score was associated with unfavorable outcomes. The conditional effect plot of the estimated risk against SOFA score upon admission categorized according to the AKIN stages showed that the risk of in-hospital mortality was highest among patients in AKIN stage 3 with a high SOFA score. In previous studies, factors related to the outcomes of adult patients with acute myocarditis remained unpredictable. Some laboratory parameters, such as interleukin 10 and Fas-ligand, were reported as valuable predictors among these patients [26]. Unfortunately, these studies were limited by small sample sizes and were not available in general clinical care. Since the symptoms of acute myocarditis developed dramatically and EMB was an invasive procedure, we included patients with acute myocarditis mainly diagnosed clinically according to previous definition [20]. In previous studies, the predictive value of viral genome detection or Dallas criteria was still controversial [27]–[29]. In this study, Dallas criteria was not associated with in-hospital mortality among patients who underwent EMB (p = 0.4). A low LVEF was an indication of depressed systolic function, while elevated CK-MB level revealed severe myocardial injury. In our series, neither LVEF nor CK-MB level upon admission was identified as a predictor of in-hospital mortality in multivariate analysis. An initial elevated cardiac enzyme and depressed systolic function were not viewed as evidence of irreversible myocardial damage. Unfavorable outcomes were associated with patients categorized as AKIN stage 3, rather than whether they received ECMO. Early intervention and aggressive therapeutic strategies, such as extracorporeal life support and optimization of hemodynamic parameters, should be performed to prevent a worsening renal function and reduce the risk of mortality. AKIN classification is an easy tool and provides us objective information of disease progression.

Our study has some limitations. As a retrospective study, we did not have the opportunity to compare the effects of different interventions. Second, EMB was not performed on all patients with symptoms that developed dramatically. Nonetheless, combining the clinical features of viral myocarditis and substantial improvement in the left ventricular function supports a clinical diagnosis of active myocarditis. Third, this study did not employ cardiovascular magnetic resonance (CMR) imaging. CMR is a valuable noninvasive diagnostic imaging tool that has proven highly effective in identifying areas of myocardial damage [30]. Further research could benefit from the use of CMR when studying patients with acute myocarditis.

In conclusion, nearly three-fifths of patients who suffered from acute myocarditis had AKI, resulting in a significant increase in mortality. The increase in mortality was associated with the progression of the patient through the stages of AKIN. Multivariate analysis identified AKIN stage 3 and elevated SOFA score as independent risk factors of in-hospital mortality. AKIN classification is a simple, reproducible, and easily applied evaluation tool capable of providing objective information related to the clinical prognosis in patients with acute myocarditis. The correlation between AKI and the prognosis of acute myocarditis should be further evaluated in future randomized controlled trials.
